# Effect of pre-partum prilled fat supplementation on feed intake, energy balance and milk production in Murrah buffaloes

**DOI:** 10.14202/vetworld.2016.256-259

**Published:** 2016-03-11

**Authors:** Shikha Sharma, Mahendra Singh, Ashwani Kumar Roy, Sunita Thakur

**Affiliations:** Division of Dairy Cattle Physiology, National Dairy Research Institute, Karnal, Haryana, India

**Keywords:** body condition score, buffaloes, dry matter intake, energy balance, milk yield, prilled fat

## Abstract

**Aim:**

To investigate the effect of pre-partum prilled fat feeding on dry matter intake (DMI), energy balance and milk production in Murrah buffaloes.

**Materials and Methods:**

Advance pregnant Murrah buffaloes were either received a dietary supplement of prilled fat at 100 g/day for 35 days pre-partum and at 150 g/day for 95 days post-partum (supplemented group [SG]) or did not receive fat supplement (control group [CG]). DMI and the yields of milk and milk component were measured. A body condition score (BCS) was recorded. Energy balance and gross feed efficiency (GFE) were calculated. DMI and BCS were recorded and milk yield (MY), fat, protein, lactose, solid not fat, energy balance were measured. The fat corrected milk yield was calculated.

**Results:**

The DMI was non-significant between groups and periods of study. BCS of buffaloes improved in the SG than CG (p<0.01). The energy intake in terms of total digestible nutrients (TDN%), TDN intake, digestible energy (DE), metabolizable energy/kg of milk, DE of milk, net energy, and GFE were higher (p<0.01) in SG during post-partum period. Crude protein intake was statistically similar in both the groups. MY was higher (p<0.01) in SG than in CG during 95 days of early lactation. Milk fat, fat corrected MY was higher (p<0.01) in SG however protein, lactose and solid not fat content did not varied between the groups. The feed efficiency of the SG was higher (p<0.01) than the CG during the post-partum period.

**Conclusion:**

It was inferred that prilled fat supplementation augments energy balance and milk production in transition Murrah buffaloes.

## Introduction

The transition period is a critical determinant of both productivity and profitability of a dairy farm. During this period, hormones and metabolites significantly alter to mobilize the body reserves to meet energy requirement of fetus and lactogenesis. Freshly calved high yielding dairy animal cannot meet energy requirements by routine feeding practices and have to mobilize body energy reserves resulting in loss of body weight which is further attenuated by the gap in feed intake and peak milk yield in early lactation [1]. The energy content of the diet can be increased during transition period by providing energy density ration through incorporation of fat in the diet of cows [2].

Various forms of bypass fat including Ca salt of fatty acid have been found effective to augment animal productivity without any adverse effect on feed intake in cows and buffaloes [3-5] without affecting rumen cellulolytic bacterial activity [6]. Liquefying a mixture of fatty acids having high saturated fatty acid content and spraying the mixture under pressure into a cooled atmosphere, results in a dried prilled fat that is inert in the rumen and does not alter rumen fermentation. Prilled fat is a bypass fat that does not affect feed intake, and is broken down in the intestine by lipase enzyme and enhances milk production performance in cows and buffaloes [7,8].

To the best of our knowledge, the information on the beneficial effect of prilled fat supplementation in relation to improvement in energy balance during transition period, if any is lacking in Murrah buffaloes. The supplementation of prilled fat at this crucial stage may improve the milk production through increase in energy balance of buffaloes. With this hypothesis, the present investigation was undertaken to measure feed intake, body condition score (BCS), energy balance, and milk production performance of Murrah buffaloes supplemented with prilled fat during the transition period.

## Materials and Methods

### Ethical approval

The experimental protocol was duly cleared by the Institute Animal Ethic Committee.

### Experimental Animals and experimental design

The experiment was conducted on 14 advanced pregnant Murrah buffaloes in the second parity at - 35 days pre-partum. The buffaloes were selected based on most probable production ability range 1903-2218 kg. Experimental buffaloes were stall fed and managed in an asbestos roof shelter with brick floor. Buffaloes were equally divided into two groups as control (CG; body weight 570-744 kg) and supplemented buffaloes (S-group; 529-760 kg) and were fed green fodder (berseem/sorghum/maize), wheat straw and concentrate mixture in the ratio of 60:40 in the morning at 9:30 am. Experimental buffaloes received prilled fat at 100 g/day during the pre-partum period and at 150 g/day for a period of 95-day post-partum. The amount of green fodder given and leftovers were weighed at weekly intervals, and dry matter intake (DMI) was calculated. The composition of concentrate mixture and chemical composition of feed is presented in the Tables-1 and 2. The buffaloes were hand milked twice a day in the morning (6 am) and evening (5:30 pm) and milk yield (MY) was recorded daily.

**Table-1:** Physical composition of concentrate mixture.

Ingredients	Percent part
Maize	33
Groundnut cake	21
Mustard cake	12
Wheat bran	20
Deoiled rice bran	11
Mineral mixture	2
Common salt	1

**Table-2:** Chemical composition of feed offered (%DM basis).

Parameters	Concentrate	Green fodder	Wheat straw
DM	91.70	16.72	90.20
OM	91.15	88.93	91.58
CP	20.20	8.68	3.11
EE	3.87	2.02	1.31
NDF	37.12	52.48	77.9
ADF	13.36	33.05	50.35
Ash	8.85	11.07	8.42

DM=Dry matter, OM=Organic matter, CP=Crude protein, EE=Ether extract, NDF=Neutral detergent fiber, ADF=Acid detergent fiber

### Sample collection and analysis

BCS was recorded by observing the fat deposition by five-point scale method with the help of an attendant [9]. Milk samples were collected and analyzed for fat, protein, lactose and solid not fat (SNF) by milkotester (M/S Meganetco). The detail of proportions of different ingredients in the diet and their nutrient compositions is given in Tables-1 and 2. Energy balance in terms of total digestible nutrients intake (TDNI), TDN%, crude protein intake (CPI), digestible energy (DE), metabolizable energy (ME), net energy (NE) and ME/kg of milk, DE/kg of milk was calculated using the following formulas:

DE (Mcal/kg) = 0.04409 × TDN (%)

ME (Mcal/kg) = 1.01 × DE−0.45

NE (Mcal/kg) = 0.0245 × TDN (%) − 0.12

Feed efficiency (FE), gross FE (GFE) and energy corrected milk was determined [10]. The fat corrected MY (FCMY) was calculated using formula = (0.4 × MY + 15 fat)/1.3 [11].

### Statistical analysis

The analysis of variance (two-way) was carried out using Sigma stat32 program. The comparison between the groups (control vs. supplemented) and periods (pre-partum, day of parturition, post-partum) was carried out and the mean and standard errors were calculated. A pearson correlation matrix among various parameters is presented in Table-3.

**Table-3:** Post-partum correlation coefficient (r) among BCS, DMI and MY.

Parameters	Correlation coefficient (r)	Significance
BCS versus DMI	0.214	p<0.05
BCS versus MY	0.315	p<0.05

BCS=Body condition score, DMI=Dry matter intake, MY=Milk yield

## Results

The postpartum body weight of SG buffaloes was significantly (p<0.01) more and the decline in body weight on day of parturition was less in comparison to CG (Table-4). The post-partum BCS score increased (p<0.01) in SG over the CG. DMI varied between the group (p<0.03) and periods (pre-partum, parturition, post-partum) of study (p<0.01). The interaction of group × period was significant (p<0.01). Buffaloes of SG consumed more DMI (p<0.01) than the CG during pre-partum period however post-partum DMI changes between the group were non-significant. TDNI was more by 2 kg/day in SG (p<0.04) than the CG (Table-5). The DE and ME were more in SG (p<0.01) than the CG. ME/kg of milk and DE of milk was higher in SG (p<0.01) as compared to CG buffaloes. The NE was significantly lower in the CG (p<0.01) than the SG. The buffaloes of SG consumed more TDN (p<0.01) than the CG, however, CPI was numerically less in SG. GFE and FE were more in SG (p<0.01) in comparison to CG. MY increased significantly (p<0.01) at 2 kg/day during different weeks of the experiment (p<0.01) in SG in comparison to CG (Table-6). Energy corrected milk was more (p<0.01) in the SG (at 2.47 kg) than the CG. The milk fat and FCMY was higher by 6% and 23% in the SG (p<0.01) as compared to CG. Milk protein, lactose and SNF contents varied non-significantly between the groups, changes in fat, protein and lactose were non-significant between week and animal (p<0.01; Table-6). BCS was positively correlated with DMI (r=0.214, p<0.01) and MY (r=0.315, p<0.01). However, no correlation was observed between DMI, MY and milk fat content (Table-3).

**Table-4:** Mean body weight, BCS and DMI during different periods of experiment in buffaloes.

Parameters	Pre-partum		Day of parturition		Post-partum	
		
	CG	SG	CG	SG	CG	SG
Body weight (kg)	652.80±20.19	655.55±38.99	598.57±19.31	611.85±38.84	584.16^a^±19.15	625.35^b^±37.70
BCS	2.84^a^±0.18	3.21^a^±0.19	2.21^b^±0.15	2.51^b^±0.19	2.35^b^±0.13	3.40^a^±0.16
DMI (kg/day)	15.16^a^±0.17	15.61^a^±0.07	14.73^a^±0.06	15.18^b^±0.08	16.68^b^±0.14	16.73^bc^±0.13

Different superscripts (a, b, c) differ (p<0.01) in a row. CG=Control group, SG=Supplemented group, BCS=Body condition score, DMI=Dry matter intake

**Table-5:** Energy intake of feed and milk energy balance in control and prilled fat supplemented buffaloes.

Particulars	CG	SG
TDNI (kg/day)	9.52^a^±0.13	11.43^b^±0.43
TDN (%)	59.43^a^±1.62	69.29^b^±0.11
CPI (kg/day)	1.86±0.08	1.77±0.09
DE (Mcal/kg)	2.62^a^±0.07	3.05^b^±0.01
ME (Mcal/kg)	2.19^a^±0.07	2.64^b^±0.01
ME/kg of milk	0.84^a^±0.22	0.99^b^±0.01
DE of milk (Mcal/kg)	1.03^a^±0.03	1.22^b^±0.80
NE (Mcal/kg)	1.34^a^±0.04	1.58^b^±0.01
GFE	0.85^a^±0.01	0.99^b^±0.02
% FE (kg milk production/100 kg DMI)	66.09^a^±1.18	77.74^b^±1.79

Different superscripts (a, b) differ (p<0.01) in a row. FE=Feed efficiency, TDNI=Total digestible nutrients intake, CPI=Crude protein intake, DE=Digestible energy, ME=Metabolizable energy, NE=Net energy, CG=Control group, SG=Supplemented group, GFE=Gross feed efficiency, DMI=Dry matter intake

**Table-6:** Mean milk yield and composition changes in control and prilled fat supplemented buffaloes.

Attributes	CG	SG
Milk yield (kg/day)	10.87^a^±0.21	12.85^b^±0.32
ECM (kg/day)	14.03^a^±0.28	16.50^b^±0.41
FCMY (kg/day)	12.96^a^±0.27	15.99^b^±0.44
Fat (%)	7.48^a^±0.09	7.93^b^±0.10
Protein (%)	3.56±0.05	3.54±0.04
SNF (%)	9.57±0.12	9.56±0.11
Lactose (%)	5.61±0.05	5.62±0.04

Different superscripts (a, b) differ (p<0.01) in a row. CG=Control group, SG=Supplemented group, ECM=Extracellular matrix, FCMY=Fat corrected milk yield, SNF=Solid not fat

## Discussion

The improvement in MY in SG could be attributed to higher ME and TDNI and is in agreement with the earlier findings in cows [4,12,13] and buffaloes [8]. The net increase in MY was higher (18%) in this study than the 10% increase in MY reported in buffaloes fed with prilled fat at 75 g/day for 45 days and 150 g/day for 75 days in early lactation [8]. This also indicates that pre-partum feeding was more effective in eliciting the higher milk production response than feeding in early lactation. Prilled fat feeding also augments MY in crossbred cows [14,15]. The higher milk fat content in SG was due to elevated saturated and unsaturated dietary fatty acids being directly incorporated in milk fat after absorption from the intestine [16]. The non-significant effect of prilled fat feeding on milk lactose, SNF, protein has also been reported by researcher in bypass fed cows and buffaloes [2,6,17,18]. The higher values of ME, TDNI, TDN%, DE and NE values in SG suggest role of prilled fat in improving the energy balance and more partitioning of nutrients to mammary gland leading to higher yields. The increased energy density of ration reduced the deleterious effect of negative energy balance as indicated by higher ME and DE/kg of milk in SG over the CG. It was also evident from higher FE and GFE observed in this study and reported earlier in lactating animals [8,18]. This could be the reason of a significant increase in BCS of SG buffaloes than the CG [17,19]. The higher pre-partum DMI intake (p<0.01) in SG than the CG and a non-significant effect on post-partum DMI intake observed in this study is in agreement with earlier reports [20,21]. The results of the present study reveal that the supplementation of prilled fat improves energy balance during advance pregnancy which in turn causes higher MY in ensuing lactation [22]. In addition to this, milk fat %, FCMY and energy corrected MY increases due to improved BCS.

## Conclusion

Supplementation of prilled fat during pre-partum (at 100 g/day) and post-partum (at 150 g/day) period resulted in a significant increase in MY and milk fat content concomitant to increase in BCS and energy balance without affecting milk protein, lactose, SNF and DMI in Murrah buffaloes.

## Authors’ Contributions

SS: Collection of milk and feed samples, daily milk recording and supervision of experimental animals. MS: Selection of animals, experimental design, data analysis and draft and revision of the manuscript. AKR: Recording of body weight and BCS. ST: Analysis of milk samples. All authors read and approved the final manuscript.

## References

[1] Ingvartsen K.L, Andersen J.B (2000). Symposium: Dry matter intake of lactating dairy cattle. Integration of metabolism and intake regulation: A review focusing on periparturient animals. J. Dairy Sci.

[2] Sirohi S.K, Walli T.K, Mohanta R.K (2010). Supplementation effect of bypass fat on production performance of lactating crossbred cows. Indian. J. Anim. Sci.

[3] Ganjkhanlou M, Rezayazdi K, Ghorbani G.R, Dehghan Banadaky M, Morraveg H, Yang W.Z (2009). Effects of protected fat supplements on production of early lactation Holstein cows. Anim. Feed Sci. Technol.

[4] Karcagi R.G, Gaál T, Ribiczey P, Huszenicza G, Husveth F (2010). Milk production, peripartal liver triglyceride concentration and plasma metabolites of dairy cows fed diets supplemented with calcium soaps or hydrogenated triglycerides of palm oil. J. Dairy Res.

[5] Shelke S.K, Thakur S.S, Amrutkar S.A (2012). Effect of feeding protected fat and proteins on milk production, composition and nutrient utilization in Murrah buffaloes (*Bubalus bubalis*). Anim. Feed Sci. Technol.

[6] Thakur S.S, Shelke S.K (2010). Effect of supplementing bypass fat prepared from soybean acid oil on milk yield and nutrient utilization in Murrah buffaloes. Indian J. Anim. Sci.

[7] Rajesh G (2013). Postpartum physiological adaptation in hormones, metabolites and milk production in crossbred cows fed with prilleded fat.

[8] Singh S (2015). Nutritional effects on metabolic profile and early lactation performance of Murrah buffaloes fed diet supplemented with prilledd fat.

[9] Wattiaux M.A (2005). Body Condition Scores.

[10] Varga G.A, Ishler V.A (2014). Managing Nutrition for Optimal Milk Components.

[11] Rice V.A (1970). Breeding and Improvement of Farm Animals.

[12] Silvestre F.T, Carvalho T.S.M, Francisco N, Santos J.E.P, Staples C.R, Jenkins T.C, Thatcher W.W (2011). Effects of differential supplementation of fatty acids during the peripartum and breeding periods of Holstein cows I. Uterine and metabolic responses, reproduction and lactation. J. Dairy. Sci.

[13] Weiss W.P, Steinberg W, Engstrom M.A (2011). Milk production and nutrient digestibility by dairy cows when fed exogenous amylase with coarsely ground dry corn. J. Dairy Sci.

[14] Bugalia I.I.L, Chaudhary J.L (2010). Effect of feeding different levels of formaldehyde treated sesame cake on nutrients intake, milk production and economic returns in lactating cross bred cows. Indian J. Anim. Sci.

[15] De Campeneere S, De boever J.L, Vanacker J.M, De Brabander D.L (2010). Reducing nitrogen excretion and soybean meal use by feeding a lower rumen degradable protein balance and protracted soybean meal to dairy cattle. Arch. Anim. Nutr.

[16] Fahey J, Mee J.F, Murphy J.J, Callaghan D.O (2002). Effects of calcium salts of fatty acids and calcium salt of methionine hydroxyl analogue on plasma prostaglandin F2alpha metabolite and milk fatty acid profile in late lactation Holstein Friesian cows. Theriogenology.

[17] Naik P.K, Saijpaul S, Sirohi A.S, Raquib M (2009). Lactation response of cross bred dairy cows fed indigenously prepared rumen protected fat - A field trial. Indian J. Anim. Sci.

[18] Tyagi N, Thakur S.S, Shelke S.K (2009). Effect of feeding bypass fat supplement on milk yield, composition and nutrient utilization in crossbred cows. Indian J. Anim. Nutr.

[19] Singh M, Sehgal J.P, Roy A.K, Pandita S, Rajesh G (2014). Effect of prilled fat supplementation on hormones, milk production and energy metabolites during mid-lactation in crossbred cows. Vet. World.

[20] Mishra S, Thakur S.S, Rakesh K (2004). Milk production and composition in crossbred cows fed calcium salts of mustard oil fatty acids. Indian J. Anim. Nutri.

[21] Theurer M.L, Block E, Sanchez W.K, McGuire M.A (2009). Calcium salts of polyunsaturated fatty acids deliver more essential fatty acids to the lactating dairy cow. J. Dairy Sci.

[22] Singh M, Roy A.K, Sharma S (2015). Augmentation of milk production by supplementing bypass fat in dairy animals. Asian J. Anim. Vet. Adv.

